# Extraction of First Permanent Molars in Children—A Comprehensive Review of History, Aim, Space Closure and Other Consequences

**DOI:** 10.3390/jcm14072221

**Published:** 2025-03-25

**Authors:** Ahmad Al Masri, Mhd Said Mourad, Christian H. Splieth, Karl-Friedrich Krey, Julian Schmoeckel

**Affiliations:** 1 Department of Pediatric Dentistry, University Medicine Greifswald, Walther-Rathenau-Str 42a, 17489 Greifswald, Germany; ahmad.almasri@uni-greifswald.de (A.A.M.); mhd.mourad@uni-greifswald.de (M.S.M.); splieth@uni-greifswald.de (C.H.S.); 2 Department of Orthodontics, University Medicine Greifswald, Walther-Rathenau-Str 42a, 17489 Greifswald, Germany; karl-friedrich.krey@uni-greifswald.de

**Keywords:** first permanent molars, orthodontics, pediatric dentistry, space closure, tooth extractions

## Abstract

Compromised first permanent molars (FPMs) in children pose major challenges for dentists and patients even in low-caries-risk populations. Whether due to caries or Molar Incisor Hypomineralization (MIH), compromised FPMs at an early age require careful treatment planning and timing of extraction, if necessary, to eventually have good space closure with healthy dentition. This comprehensive review explains the concept of early extraction of the FPMs in children and lists the results of published studies and systematic reviews regarding spontaneous or orthodontical space closure after the extraction of an FPM, including its consequences in the long term. In general, the majority of the studies confirm that spontaneous space closure after the early extraction of the maxillary FPM could be achieved if the extraction is performed before the eruption of the second permanent molar (SPM). On the other hand, space closure in the mandible is possible in case of optimal timing of extraction and supporting co-factors, but in practice, it would most probably require orthodontic treatment. The decision to retain or extract compromised FPMs must always be made on an individual basis, taking into account all relevant factors and the long-term effects on the entire stomatognathic system. Early prophylaxis to prevent caries and early management of MIH-affected FPMs should, however, be the first-line approach.

## 1. Introduction

Dental caries is one of the most common diseases worldwide, which has been the focus of a great number of measures and prevention programs, successfully resulting in a significant caries decline in permanent teeth in children across Europe in 2019 compared to 1990 [1]. However, due to the polarization of high-caries-risk individuals in low-caries-risk population, the first permanent molars (FPMs) are the teeth mostly affected by caries in adolescents [2,3,4]. The reasons are early eruption, anatomical structure, difficulties in daily hygiene during eruption at young age and/or Molar Incisor Hypomineralization (MIH) [5]. The dental treatment of compromised FPMs in children is very challenging, as these teeth are often not fully erupted, are difficult to isolate, and have large pulps that are often irreversibly damaged because of wide open dentinal tubules. This would frequently lead to endodontic treatment, which is challenging due to open apices in teeth with incomplete root development, necessitating more complex approaches such as revascularization techniques, apexification, apical plug, or extraction.

In addition, there are challenges beyond the tooth level, as these patients are relatively young and mostly not compliant for longer sessions to perform such complex dental treatments. Therefore, the extraction of compromised FPMs is a treatment option adopted by many dentists aiming to give their patients the opportunity to have a healthy dentition in the long term. This is performed to avoid the restorative cycle potentially leading to an extraction at a later age with possibly more disadvantages.

Over the past decades, multiple studies aimed to examine the extraction of the FPMs and its consequences, especially on space closure, with many systematic reviews in this area. As each systematic review focused on a very specific PICO question (P: Participants, I: Intervention, C: Control, and O: Outcome), each of these reviews included different papers. The main advantage of a comprehensive review is to include as many related papers as possible to attain a comprehensive collection of evidence on the topic. Therefore, this comprehensive review addresses this topic, starting from the role of the FPMs from an orthodontic point of view and the history of extraction of the FPMs, as well as the potential of (spontaneous) space closure, its predicting factors, and/or the need for orthodontic intervention for optimal results.

## 2. The Role of FPMs in Growth and Development

The anatomical peculiarity of the FPMs lies in their complex structure. They typically have three roots in the upper jaw, while the lower FPMs usually have two roots. Their occlusal surface is characterized by a pronounced cusp–fissure morphology, which is optimized for the efficient comminution of food. Compared to the second permanent molars (SPMs), the FPMs show a more complex crown structure and less variability in root morphology, which underlines their particular importance for chewing function and the stability of the dentition [6,7].

When they erupt, the FPMs are angulated distally and the occlusal plane is initially very steep. In the course of development, approximately between the 4th and 20th year of life, the occlusal plane flattens and the FPMs straighten [8].

The FPMs are considered the key to occlusion in orthodontics, a concept that goes back to Edward Angle. The FPMs play a decisive role in Andrews’ “Six Keys of Normal Occlusion”, in which the mesial cusp of the upper FPM occludes with the lower transverse fissure, the distal marginal ridge is in contact with the mesial marginal ridge of the lower SPM, and the mesiopalatal cusp is in the pit of the lower FPM [9].

Slavicek emphasizes the special significance of the FPMs in his work: “With the eruption and setting of the FPM and anterior teeth, a completely new morphological principle enters the system. Genetically predetermined, the new morphology determines the functional events and the neuromuscular system must adapt to the new supply” [10].

The functions of the FPMs in the masticatory system are diverse. They provide vertical and transversal support for the occlusion, stabilize the second physiological bite elevation, and are important for the retral protective function as well as bolus transport and food chewing. They also protect the surrounding soft tissues such as the cheek and tongue.

One particular aspect is the role of the FPMs in force transmission and their neurological significance. The maxillary FPM plays a decisive role in the transmission of chewing pressure via the zygomatic arch into the skull. In addition, the FPMs have a very large cortical representation in the cerebral cortex, which underlines their importance for the sensitivity of the dentition [11].

Finite element models have shown that even when the gap is closed after extraction of the FPMs, the maxillary force transmission is altered: “The occlusal forces were transferred through maxillonasal, maxillozygomatic, and maxillopterygoid stress trajectories. The mesial displacement of the molars may weaken the role of maxillopterygoid stress trajectory while strengthening the role of maxillonasal stress trajectory” [12].

## 3. Long-Term Evaluation of Conservative Treatments of Compromised FPMs

Preventing tooth loss is one of the most important tasks of (pediatric) dentists worldwide. However, the aspect of sustainability and cost-effectiveness should not be forgotten. This raises the question of the long-term prognosis of dental treatments to preserve compromised FPMs, especially if the treatments are not performed under optimal conditions due to limitations such as the cooperation of the child or difficulties in isolation. A published article in community health research after 4 years of observations of the outcome of over 14 million dental fillings showed a survival/success rate varying between 55.8 and 74.8%, while the success rate of over 500,000 endodontic treatments after 3 years of observation reached 84.3% [13]. Due to the complexity of regenerative endodontic treatment in immature permanent teeth, there is very little evidence of the success of different approaches in the long term [14]. Slightly better success rates are reported for single crowns, reaching 94% over 6 years of observation [15]. Although those success rates can be considered satisfactory, it is important to anticipate lower success rates in children if assessed after more than 6 years. This would suggest possible repeated or even continuous treatment for FPMs affected at an early stage and a possible need for extraction at mid-age, where implants might be necessary for the preservation of bone and improvement of chewing. However, the long-term assessments of dental implants show survival rates varying between 73 and 95.5%, depending on type of implant and the experience of the practitioner. Therefore, it must be considered, when planning the preservation of compromised FPMs, that after entering the restoration cycle, multiple treatments and interventions are probably necessary over the course of life, if the aim is tooth preservation for more than 30–40 years. The other aspect to consider is cost-effectiveness; a cost-effectiveness analysis within the German healthcare system showed that it is more cost-effective to extract compromised FPMs at a young age and aim for space closure with or without orthodontics than conservative treatments over 50 years, especially if more than one FPM are compromised [16]. Another study on the cost-effectiveness of retaining or extracting compromised FPMs in the United Kingdom (UK) concluded that retaining these compromised teeth is more expensive than extraction, considering that general anesthesia is not needed [17].

## 4. History of Extraction of FPMs in Children

Historical studies on the development of caries over the centuries suggest a very low prevalence of caries until the middle of the nineteenth century, where industrial development and the change in diet probably led to a rapid increase in caries prevalence. In those times, when caries prevalence was low, the first available mention of the extraction of the FPMs in children in the literature was from Maclean in 1857, citing the recommendations of Mr. Fox in 1803 for the idea of the “systematic removal of the four permanent first molars at an early period, in a large majority of cases, when incipient caries is present” [18]. The advantages of the suggested recommendations were as follows:

(1) The prevention and correction of the simpler forms of irregularities in the easiest and most desirable way, in a great majority of cases, without the aid of mechanical means; in all, in such a manner as least to disfigure the appearance of the mouth.
(2) The promotion of a healthier state amongst the remaining teeth; the prevention (probably) of caries, certainly an increase in the facility of treating it.
(3) The prevention of the distressing, in some cases even very serious, symptoms which frequently accompany the development of the wisdom teeth in corroded arches, and a material diminution in the chance of the formation of sinuses in after-life.

Later on in the 20th century, with increased caries prevalence, there was increased attention towards restorative materials and root canal treatments, but a return to situations of very low caries prevalence in developed countries raises the question of having natural healthy dentition through the extraction of compromised FPMs, as reflected in the rapid increase in studies on this topic.

## 5. Space Closure After FPM Extraction: A Systematic Search of Studies

In order to evaluate the consequences of FPM extraction with respect to the resulting space in the arch, a systematic search was conducted to provide an overview of the possible outcomes.

### 5.1. Methods of the Literature Search and Research Question

**Figure 1:**
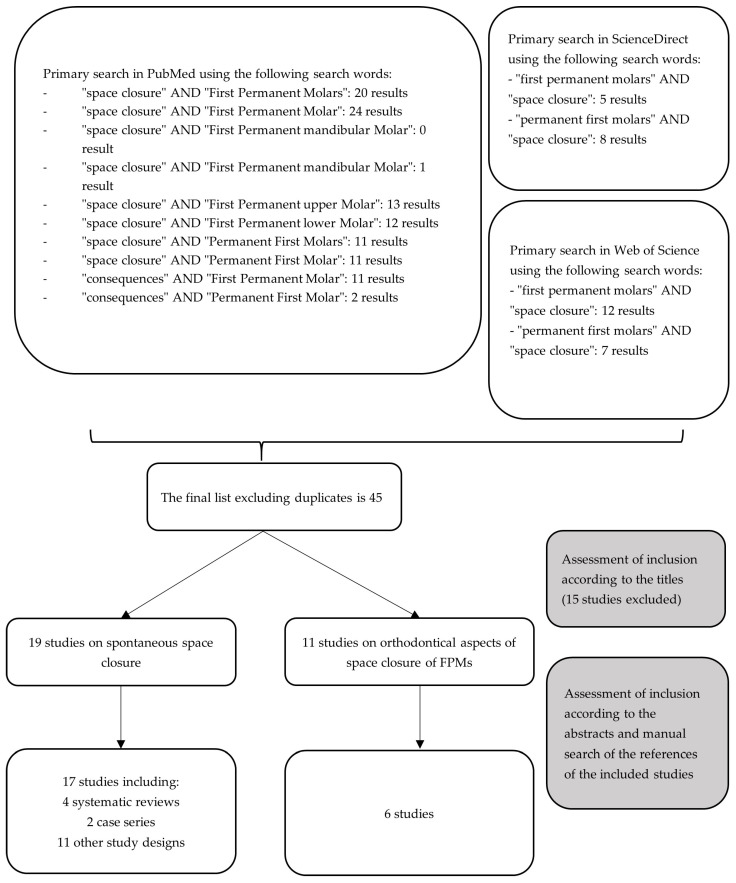
Flow chart of the systematic literature search for this comprehensive review.

The research question focused on investigating space closure after the extraction of the FPMs using the PICO framework (P: children under 18 years of age, I: extraction of one or more FPMs with or without subsequent orthodontic treatment, C: no comparator group was specified, O: space closure analyzed visually or radiographically after the complete eruption of the second permanent molar (SPM)). All published study types in English or German were included, with no restrictions on the date of publication. The databases of PubMed, ScienceDirect, and Web of Science were searched for relevant studies using specific search terms that were used in similar studies (Figure 1).

### 5.2. Spontaneous Space Closure After FPM Extraction

The final included studies were thoroughly read for data extraction by one experienced researcher, while the correctness of the extracted data was controlled by another experienced researcher. An overview of the studies included in this review with their main conclusions is presented in Table 1, while the five included systematic reviews are summarized in Table 2.

### 5.3. Space Closure After FPM Extraction from an Orthodontic Point of View

The late extraction of the first permanent molars presents a significant orthodontic challenge, particularly in achieving effective space closure. Treatment planning requires the consideration of various factors, including the patient’s age, alveolar bone topography, periodontal health, and overall orthodontic considerations. When unfavorable prognostic factors are identified, alternative therapeutic approaches may need to be explored [35].

Jacobs et al. [36] assessed the mesialization of the SPM as a strategy for space closure following FPM extraction. The study involved 35 patients and demonstrated that bodily tooth movement was achieved with minor tipping or uprighting. However, notable side effects included the retrusion of incisors and posterior displacement of soft tissues, which resulted in alterations to the patient’s profile. These findings underscore the importance of evaluating anchorage systems to minimize adverse effects during treatment.

Raveli et al. [37] emphasized the challenges of space closure in orthodontics, such as extended treatment duration, patient discomfort, and concerns regarding tissue tolerance and stability. Their case report demonstrated the use of T-loops as an effective mechanical approach to control space closure. This technique facilitated precise tooth translation while minimizing abrasion, highlighting the importance of selecting appropriate mechanics for optimal outcomes.

Cardoso et al. [38] examined the influence of the loss of the FPMs on the duration of the orthodontic treatment. Patients undergoing space closure without skeletal anchorage experienced longer treatment times (mean time of 44.7 months) compared to those without tooth loss (mean time of 22.5 months). Prolonged treatment duration was associated with factors such as multiple tooth extractions, involvement of both arches, and missed appointments. These results emphasize the need for patient compliance and consideration of skeletal anchorage to optimize treatment efficiency.

Orthodontic space closure after the extraction of the FPMs can influence the angulation and positioning of adjacent teeth. A longitudinal analysis by Langer et al. [39] evaluated 152 panoramic radiographs of patients with or without FPM extractions. The study found significant improvements in the angulation of the maxillary second and third molars (*p* < 0.001) in the extraction group. Improvements in mandibular third molar inclination were observed (*p* < 0.01). However, vertical positional changes in the mandible were not statistically significant. These findings suggest that extraction of the FPMs may enhance the eruption space and prognosis for the third molars in the maxilla.

A finite element analysis conducted by Sana et al. [12] investigated the biomechanical effects of space closure following maxillary first molar extraction. The study revealed changes in occlusal stress distribution, with increased stress observed in the maxillonasal and maxillozygomatic trajectories. Additionally, mesial displacement of the molars appeared to weaken the maxillopterygoid stress trajectory while strengthening the maxillonasal trajectory. These findings highlight potential implications for long-term occlusal stability.

## 6. Patient-Related Consequences of FPM Extraction

Most studies found in the literature regarding the consequences of the extraction of FPMs in children are mainly concerned with space closure or occasionally with angulations and rotations of the neighboring teeth. However, there are many other factors that should be considered, and most are not included as factors in the assessment of consequences.

Chewing is an important part of food intake. Only by reducing food into small particles can they be swallowed. Chewing efficiency, therefore, plays an important role in food intake and digestion [40]. Some studies show that there may also be a relationship between occlusion and chewing efficiency in children [41,42]. The influence of the removal of an FPM in the mixed dentition on mastication is still unknown. Furthermore, it is unclear whether differences in masticatory efficiency compared to “normal” dentition are detectable after space closure by the SPM.

Medical treatments should not only achieve an improvement in a person’s physiological condition, but also consider the accompanying psychological and social changes [43,44]. In dentistry, oral health-related quality of life in adults and children has recently been increasingly highlighted, for which several survey tools have been developed [45,46]. The success of a treatment or concept is almost always determined by the practitioner alone. However, the well-being and satisfaction of those treated and the fulfillment of expectations are aspects that influence the evaluation of a treatment and should be considered when choosing a treatment option. The consideration of the so-called “patient-related outcome (PRO)” is, therefore, increasing in the literature in order to further optimize medical care [47,48,49]. However, no studies assessing oral health-related quality of life of children after the extraction of the FPMs and/or after space closure were found in the literature.

Overall, it must be noted that these aspects, such as chewing efficiency, oral health-related quality of life, patient/parent satisfaction, and dentists’ treatment preferences regarding the extraction of the FPMs, have been little researched although they are of great importance. This is especially true when patients’ interest is taken into account, as it should be the standard in modern patient–doctor relationship. This shows an unfortunate gap in research and the necessity for further scientific evaluation efforts for better clinical care.

## 7. Discussion and Clinical Recommendations

The extraction of the FPMs in children is a complex interdisciplinary field in dentistry. The aspect of spontaneous space closure of the SPM after the extraction of a FPM has been investigated in various countries and for over a decade (Table 1). Interestingly, most clinical studies have a similar methodological concept (retrospective cohort studies), which is a more convenient and time-efficient approach than prospective (controlled) clinical trials in the reporting of long-term consequences. Overall, most studies yield similar findings, showing that the extraction of an FPM in the maxilla has a far better chance for spontaneous space closure than in the mandible. Additionally, the timing appears to be a crucial factor, which is depicted by Demirjian’s development stage of the SPM rather than the patient’s age. This has been proposed as the main influencing factor, which obviously correlates with the dental age of the patient and helps to differentiate between early and late extraction of the FPM (Table 1 and Table 2).

The results of this comprehensive review confirm that a good number of studies support the concept of early extraction of compromised FPMs to have a chance of spontaneous space closure through the SPM. This approach is definitely a viable and good treatment option, as it is a relatively simple dental procedure and is likely cost-effective in the long run [16], especially if more than one FPMs are compromised and spontaneous space closure is achieved. Nonetheless, it has to be considered that, especially in cases of multiple compromised FPMs to be extracted, the treatment may require sedation methods or even general anesthesia due to the age and limited cooperation of children.

Generally, patient-related factors play a large role in this treatment option. At first, it may sound contradicting to extract permanent molars (with bad prognosis) for a better oral health-related quality of life, but parallel to good prevention, this concept is a viable thought-provoking approach as it may not only improve the quality of life in the short run but also offers the chance for a healthy permanent dentition in the long term with less dental visits and dental treatment need. One has to deeply consider that alternatives such as endodontic/root canal treatment at a young age may not even be successful. Moreover, the option of providing implants early in children, or sometimes in young adults, is not available or not recommended. Complex prosthetic treatments like crowns and bridges are also not useful at this age. The option to perform an autotransplantation, e.g., of the third molar to the position of a FPM, is usually a matter of late extraction during late adolescence (≥16 y) to early adulthood. Still, this approach to the extraction of FPMs also has certain risks, advantages, and disadvantages, which need to be considered on an individual basis.

Especially in countries with high caries prevalence, low financial family background, and/or reduced/no dental reimbursement systems, the extraction of the FPMs during mixed dentition is probably an even more pragmatic and efficient option as it is relatively cheap and quick (short clinical procedure and less dental visits needed), and still has good chances for future health under the correct circumstances. This can be deduced from the origin of the clinical studies shown in Table 1 [19,21,22,26]. However, this approach may also be beneficial for the high-risk population within a society with low caries prevalence.

Based on this comprehensive review, the following clinical recommendations are proposed:

- Prophylaxis to prevent caries and early treatment of MIH-affected FPMs should be the first-line approach to minimize the risk of later poor prognosis.
- An early interdisciplinary approach involving (pediatric) dentists, orthodontists, and oral surgeons should be taken into consideration when the prognosis of the FPMs in children is poor in order to leave time for decision making and planning. An orthopantomogram (OPT) at an age of around 8 years is highly advised in addition to clinical intra- and extra-oral examinations.
- Decisions for the extraction of FPMs in children should always be made on an individual basis with informed consent and should consider patient-related factors with long-term oral health in mind.
- Spontaneous space closure of the SPM after the extraction of a FPM is likely in the maxilla (~90%) when performed before the eruption of the SPM (radiographically at Demirjian stages D-G which reflect a wider time span: usually until the age of 11.5 y).
- Spontaneous space closure of the SPM after the extraction of a FPM in the mandible is possible (max. ~50%) when performed at the ideal time point (Demirjian stage E) and in case of further beneficial co-factors (e.g., the presence of third molars and mesial angulation). In real life, this is not always easy to achieve.
- Late extraction of FPMs in the mandible is a very likely clinical situation that requires orthodontic intervention, which may lead to highly successful results but with an increase in the complexity and the duration of the orthodontic treatment.

## 8. Conclusions

Early extraction of compromised FPMs (before 11.5 years and before the eruption of the SPM) can lead to long-term healthy dentition. Spontaneous space closure after the extraction of the FPMs in the maxilla is less dependent on optimal timing, while the extraction of the FPMs in the mandible is accompanied more frequently by the need for orthodontic treatment. The decisions for the extraction of the FPMs should always be made on an individual basis, including careful diagnosis and orthodontic and surgical consultation, with enough time to plan and consider the optimal timing taking into account the Demirjian’s development stage of the SPMs and their angulations, the presence of the third molars, general orthodontic treatment need, long-term effects on the entire stomatognathic system, etc. Nevertheless, prophylaxis to prevent caries and early treatment of MIH-affected FPMs should be the first-line approach.

## Figures and Tables

**Table 1:** Overview of the included 13 retrospective, cross-sectional studies and case reports regarding spontaneous space closure after FPM extraction in children.

Study	Country of Recruitment	Type of Study	Number and Age of Participants	Number and Type of Included Teeth	Other Exclusion Criteria	Method of Assessment of Space Closure	Main Conclusion (Predicting Factors and Optimal Extraction Timing)
Bakkal et al., 2024 [19]	Republic of Türkiye	Retrospective cohort study	29 children aged 7–14 y (mean age of 10 y) with one FPM extracted, selected out of 406 panoramic x-rays	Early extraction up to 12 yo (*n* = 15) vs. late extraction > 12 yo (*n* = 14), 13 upper FPMs, 16 lower FPMs	Patients with orthodontic treatment post extraction.	Panoramic radiographs 0–3 months before and 12–24 months after extraction	The developing dentition was affected by the extraction of the FPM depending on the extraction timing, particularly in the mandible. The ideal extraction time must be considered, keeping in view the dental age of children, and planned according to the calcification grades of SPMs for conducive outcomes.
Brusevold et al., 2022 [20]	Norway	Retrospective cohort study	27 children, mean age of 8.7 y at extraction, duration of 3.2 years for mean follow-up (1.1–6.3 y)	90 FPMs: 47 upper FPMs, 43 lower FPMs, 16 children had all 4 FPMs extracted	Patients with orthodontic treatment post extraction.	Radiographs and clinical examination	For maxillary extractions, spontaneous space closure can be anticipated (100%), while mandibular FPM may need orthodontic space closure (51.6% spontaneous space closure). When judging the upper jaw separately, 22 children had good results and one child had an acceptable result. For the lower jaw, six children had good results, eight children had acceptable results and eight children had non-acceptable outcome.
Ciftci et al., 2021 [21]	Republic of Türkiye	“Cross-sectional” based on retrospective patient file identification	133 patients, age at extraction of FPMs was 9.4 y; mean age at assessment of post-extraction radiograph was 12.7 y	177 mandibular FPMs with the full eruption of the SPM for assessment post extraction	- Patients with orthodontic treatment post FPM extraction. - Missing or extracted teeth other than FPM. - Special health care needs. - History of oral pathology.	Radiographs and clinical examination	Of the 177 mandibular SPMs, 93 (52.5%) exhibited successful space closure in the mandibular arch. The presence of the 3rd molar was correlated with successful spontaneous space closure. The developmental stage of the SPM was not found to be a statistically significant factor for spontaneous space closure. Furthermore, the angulation of the SPM had no relationship with spontaneous space closure in the mandibular arch. The development of the 3rd molars should be considered for spontaneous space closure following FPM extraction. Interdisciplinary collaboration is needed to facilitate the consequences of early FPM extraction.
Demir et al., 2020 [22]	Republic of Türkiye	“Cross-sectional” based on retrospective patient file identification	15 patients with extraction of the FPM in late stages development of the SPM (after Demirjian’s stage E) and successful spontaneous space closure assessed through the presence of contact between the teeth without spaces. Mean age was 12.8 y at extraction.	21 FPMs	Patients with orthodontic treatment post extraction.	Radiographs and clinical examination using a modified scale from Teo et al., 2013 [23]	All Spontaneous space closure after the late extraction of the FPM (after Demirjian’s stage E) can be achieved in the upper jaw. In the lower jaw, extraction can be performed considering the following: Long-term prognosis of restored PFM tooth; The dental age of the patient; Distal inclined eruption angle of the PFM; Type of malocclusion; Current degree of crowding; The presence of the permanent third molar tooth, the cases that showed ideal space closure (14%) were in the maxilla.
Elhussein and Möller 2007 [5]	Sweden	Retrospective cohort study	27 children with mean age of 8.2 years at extraction (5.6–12.7 y). Follow-up for 5.7 years (3.8–8.3 y)	79 FPMs in total, with 45 upper FPMS and 34 lower FPMs	Patients with orthodontic treatment post extraction.	Each case was followed after individual indications, and the development documented by OPT, casts, photographs, and/or bitewings.	About two-thirds of the children had good spontaneous space closure after the extraction with no or minor remaining gaps. Favorable spontaneous space reduction and development of the permanent dentition positioning can be expected without any intervention in the majority of cases if the extractions are performed prior to the eruption of the SPMs.
Nordeen et al., 2022 [24]	USA	Retrospective cohort study	162 patients (5–15 y)	138 maxillary and 168 mandibular quadrants	- Patients with orthodontic treatment prior to FPM extraction. - Missing or extracted teeth other than FPMs. - Special health care needs.	Radiographs (using Patel et al., 2017, toolkit) [25]	Chronological age and SPM developmental stage were the primary predictors for successful substitution of the extracted FPM with the SPM. The mandibular quadrants demonstrated an overall success rate of 51%. The probability of success (80%) in the mandibular arch was observed at the age of 8 y or at SPM Demirjian’s stage D. The maxillary quadrants demonstrated an overall success rate of 82%. The probability of success (91%) in the maxillary arch was observed between 8 and 10 y or between SPM Demirjian’s stages D and E.
Patel et al., 2017 [25]	United Kingdom	Retrospective cohort study	81 children with extracted FPM Mean age at extraction was 9.6 y (6–14.5 y)	148 maxillary and 153 mandibular quadrants	- Orthodontic treatment with no pretreatment record available. - Craniofacial syndromes and anomalies of eruption. - Extraction or hypodontia of other permanent teeth - No preoperative OPT	Visual examination, study models, or radiographs as success/failure	Spontaneous closure occurred in 89.9% of the maxillary and 49.0% of the mandibular quadrants. The mesial angulation of the developing SPM and the presence of the third molar have both statistically and clinically higher chances for space closure in the mandibular arch.
Sabbagh et al., 2024 [26]	Saudi Arabia	Retrospective cohort study	73 children (7–13 y; mean age at extraction: 9.5. ± 1.5 y Mean age at assessment: 14.20 ± 1.6 y	112 FPMs	- Orthodontic treatment. - Extraction of teeth other than FPM. - No radiographs at extraction. - Presence of SPM at extraction or absence of SPM at assessment.	Comprehensive Cast and radiograph assessment using ABO score	The direction of the SPM long-axis and its (early) developmental stage are key indicators of the favorable outcome pattern of spontaneous space closure after FPM early extraction. Early SPM development stage increased the probability of space closure between the SPM and second premolar.
Serindere et al., 2019 [27]	Republic of Türkiye	Retrospective cohort study	55 patients (mean age of 13.75 y at evaluation)	83 FPMs extracted between 8 and 13 y, with at least 2.5 y follow-up after the extraction	Patients with no assessable OPT	- Radiographs (Demirjian’s stage) - Clinical according to Teo et al., 2013 [23]	Spontaneous space closure according to the tooth type was as follows: #16: 55.6%/#26: 50%/#36: 38.5%/#46: 40%. Space closure in the region of bilateral extraction of the lower and upper FPMs was found to occur at the rates of 16.7% and 50%, respectively. Favorable space closure and development of SPM is expected to occur even without orthodontic treatment, although it does not always end up with satisfactory results.
Teo et al., 2013 [23]	United Kingdom	Retrospective cohort study	63 patients with at least 2 extracted FPMs (55 patients: all 4 FPMs)	236 FPMs extracted under GA 5 years before assessment	Patients with orthodontic treatment post extraction	- Radiographs (Demirjian’s stage) - Clinical assessment	Upper and lower arches yielded significantly different results, with 92% of all upper extractions resulting in complete space closure regardless of SPM development stage. Only 66% of lower FPMs extracted at SPM stage E had complete space closure, and no significant relationship was found between lower SPM development stage and its subsequent space closure.
Teo et al., 2016 [28]	United Kingdom	Retrospective cohort study	66 patients (mean age of 9.2 y at extraction and 13.8 y at follow-up)	127 lower FPMs extracted under GA 5 years before assessment	Patients with orthodontic treatment post extraction	- Radiographs (Demirjian’s stage) - Presence of molar and angulation - Clinical assessment	Only 58% of lower FPMs extracted at the ‘ideal time’ (SPM development at Demirjian’s stage E) had complete space closure. The best outcomes resulted from a combination of SPMs not at Demirjian’s development stage G, together with the presence of mesial angulation of the SPM and the presence of the third permanent molar, where 85% of those cases had complete space closure.
Elhussein and Jamal 2020 [29]	United Kingdom/Sudan	Case report	2 children Pat 1: 15 y (delayed extraction of all FPMs) Pat 2: 9 y (timely extraction of all FPMs)	All FPMs in the two cases	Not applicable	Radiographs and clinical examination/photos	While the case of delayed extractions required orthodontic treatment for space closure, the case of early extractions had spontaneous space closure without orthodontic intervention. However, the case of spontaneous space closure had an orthodontic treatment need due to crowding, which could have been directly dealt with at the extraction time. An orthodontic consultation around the age of 8 before the early extraction of FPMs is recommended.
Sabri 2021 [30]	Lebanon	Case series of 10 pat. with follow-up time up to 25 years (partial extractions as adults)	Children Pat 5: 16 y (Ex #16) Pat 8: 13 y (Ex #26) Pat 9: 12 y (Ex #46) Pat 10: 9 y (Ex all 4 FPMs)	7 FPMs with orthodontic treatment post extraction	Not applicable	Radiographs and clinical examination/photos	A well-coordinated multidisciplinary approach can facilitate the orthodontic treatment of extracted permanent first molars and achieve rewarding outcomes. Spontaneous space closure is possible, but when extraction is performed late, in adjunction with orthodontic treatment, good results are possible.

FPMs: first permanent molars; OPT: orthopantomogram; SPMs: second permanent molars; y: years old.

**Table 2:** Overview of the included four systematic reviews regarding spontaneous space closure after FPM extraction in children.

Study	Characteristics for Inclusion	Intervention	Types of Included Studies	Special Considerations	Number of Included Studies	Results of Spontaneous Space Closure in the Maxilla	Results of Spontaneous Space Closure in the Mandible	Other Conclusions
Saber et al., 2018 [31]	- Children aged 5–15 y at extraction - No orthodontic intervention	Extraction of FPM (any cause other than orthodontic reason)	clinical trials, and case–control, cross-sectional, and cohort studies	- Meta analysis - STROBE checklist	11 (3 of which regarding spontaneous space closure)	33.3–94%	50–75%	Extraction space was closed mostly by the SPM rather than by the second premolar. Space closure was more likely to occur in the maxilla than in the mandible, but the difference was not statistically significant (odds ratio 2.06, 95% confidence interval 0.46–9.28; *P* = 0.35). The ideal time of extraction is at Demerjian stage E of the SPM. It appears that the studies included in this review have too many weaknesses to draw sufficient evidence.
Hamza et al., 2024 [32]	- Children < 18 years old - No orthodontic intervention	Extraction of FPM before the eruption of SPM	clinical trials and cohort studies	- Meta analysis - Risk of bias using Joanna Briggs Institute’s tool for prevalence studies	15	85.3% (73.7–92.3%) including 665 teeth	48.1% (34.5–62.0%) including 1095 teeth	Space closure was 61.6% (50.2%, 71.9%), including 1787 teeth. Extraction of the mandibular FPMs before 10 y and at the Demirjian’s stage E of the mandibular SPM and the presence of the mandibular lower third molar is associated with increased odds of mandibular spontaneous space closure, while rotations of the mandibular molar and premolars are often seen. However, great uncertainty persists, due to the increased risk of bias of the included studies.
Eichenberger et al., 2015 [33]	- No orthodontic intervention	Extraction of FPM (any cause other than orthodontic reason)	RCTs, cohort studies, case reports	- Meta analysis - Grade of evidence was assessed	6	72% (95% CI: 63%; 82%) Age < 8 => 69% Age 8–10.5 => 80% Age 10.5–11.5 => 55% Age > 11.5 => 56%	48% (95% CI: 39%; 58%) Age < 8 => 34% Age 8–10.5 => 50% Age 10.5–11.5 => 59% Age > 11.5 => 44%	Extractions of the maxillary FPMs between 8 and 10.5 y tended to show more favorable clinical results. The analyzed data on mandibular FPM extraction demonstrated that extractions performed between 8 and 11.5 y provided significantly better results than at younger or older ages.
Alqanas et al., 2024 [34]	- Children aged 5–15 y at extraction - No orthodontic intervention	Extraction of FPM due to caries or hypomineralization	clinical trials, case–control studies, cross-sectional studies, cohort studies, and case series	Newcastle–Ottawa scale and IHE Quality Appraisal Checklist	13	52–94%	39–89%	Factors that could improve the outcome of spontaneous space closure: Optimal age: 8–10 years; Demirjian’s stages E and F; Mesial angulation of SPMl Presence of third permanent molars. The included studies had low- to moderate-level of evidence. Early assessment of SPM developmental stage and inclination and the presence of third molars are essential for enhancing the likelihood of successful spontaneous space closure following FPM extraction in children.

## Abbreviations

The following abbreviations are used in this manuscript:

FPM	First Permanent Molar
GA	General Anesthesia
MIH	Molar Incisor Hypomineralization
OPT	Orthopantomogram
RCT	Randomized Clinical Trial
SPM	Second Permanent Molar
y	Years old

## References

[1] W.H.O.: WHO/Europe Calls for Urgent Action on Oral Disease as Highest Rates Globally Are Recorded in European Region. https://www.who.int/europe/news/item/20-04-2023-who-europe-calls-for-urgent-action-on-oral-disease-as-highest-rates-globally-are-recorded-in-european-region#:~:text=The%20report%20outlines%20progress%20in,teeth%20in%20this%20age%20group.

[2] Al-Samadani K.H., Ahmad M.S. (2012). Prevalence of first permanent molar caries in and its relationship to the dental knowledge of 9–12-year olds from jeddah, kingdom of saudi arabia. ISRN Dent..

[3] Batchelor P.A., Sheiham A. (2004). Grouping of tooth surfaces by susceptibility to caries: A study in 5–16 year-old children. BMC Oral. Health.

[4] Lo E.C., Evans R.W., Lind O.P. (1990). Dental caries status and treatment needs of the permanent dentition of 6–12-year-olds in Hong Kong. Community Dent. Oral. Epidemiol..

[5] Jalevik B., Moller M. (2007). Evaluation of spontaneous space closure and development of permanent dentition after extraction of hypomineralized permanent first molars. Int. J. Paediatr. Dent..

[6] Genaro L.E., Ferreira G., Conte M.B., de Almeida Gonçalves M., Capote T.S.O. (2019). Morphological Differences between the First and Second Permanent Upper Molars. J. Morphol. Sci..

[7] Yamunadevi A., Pratibha R., Rajmohan M., Mahendraperumal S., Ganapathy N., Srivandhana R. (2021). First Molars in Permanent Dentition and their Malformations in Various Pathologies: A Review. J. Pharm. Bioallied Sci..

[8] Bathia S., Leighton B. (1993). A Manual of Facial Growth of Longitudinal Cephalometric Growth Data.

[9] Andrews L.F. (1972). The six keys to normal occlusion. Am. J. Orthod.

[10] Slavicek R. (2000). Das Kauorgan: Funktion und Dysfunktion.

[11] Oda M., Yoshino K., Tanaka T., Shiiba S., Makihara E., Miyamoto I., Nogami S., Kito S., Wakasugi-Sato N., Matsumoto-Takeda S. (2014). Identification and adjustment of experimental occlusal interference using functional magnetic resonance imaging. BMC Oral Health.

[12] Sana S., Kondody R.T., Talapaneni A.K., Fatima A., Bangi S.L. (2021). Occlusal stress distribution in the human skull with permanent maxillary first molar extraction: A 3-dimensional finite element study. Am. J. Orthod. Dentofac. Orthop..

[13] Rädel M., Walter M. (2019). Understanding and improving care: Use of routine data. Dtsch. Zahnarztl. Z. Int..

[14] Sabeti M., Ghobrial D., Zanjir M., da Costa B.R., Young Y., Azarpazhooh A. (2024). Treatment outcomes of regenerative endodontic therapy in immature permanent teeth with pulpal necrosis: A systematic review and network meta-analysis. Int. Endod. J..

[15] Ploumaki A., Bilkhair A., Tuna T., Stampf S., Strub J.R. (2013). Success rates of prosthetic restorations on endodontically treated teeth; a systematic review after 6 years. J. Oral Rehabil..

[16] Elhennawy K., Jost-Brinkmann P.G., Manton D.J., Paris S., Schwendicke F. (2017). Managing molars with severe molar-incisor hypomineralization: A cost-effectiveness analysis within German healthcare. J. Dent..

[17] Sanghvi R., Cant A., de Almeida Neves A., Hosey M.T., Banerjee A., Pennington M. (2023). Should compromised first permanent molar teeth in children be routinely removed? A health economics analysis. Community Dent. Oral Epidemiol..

[18] Samuel Maclean E. (1857). Improved Forceps, &c. Am. J. Dent. Sci..

[19] Bakkal M., Yilmaz B., Kaya M.S., Unver T., Kinay Taran P., Ozdemir S. (2024). Timing for extraction of permanent first molars in school aged children: A pilot study. J. Clin. Pediatr. Dent..

[20] Brusevold I.J., Kleivene K., Grimsoen B., Skaare A.B. (2022). Extraction of first permanent molars severely affected by molar incisor hypomineralisation: A retrospective audit. Eur. Arch. Paediatr. Dent..

[21] Ciftci V., Guney A.U., Deveci C., Sanri I.Y., Salimow F., Tuncer A.H. (2021). Spontaneous space closure following the extraction of the first permanent mandibular molar. Niger. J. Clin. Pract..

[22] Demir P., Aydoğdu H. (2020). Ideal Spontaneous Space Closure After Late Extraction of Permanent First Molar Teeth. Cumhur. Dent. J..

[23] Teo T.K., Ashley P.F., Parekh S., Noar J. (2013). The evaluation of spontaneous space closure after the extraction of first permanent molars. Eur. Arch. Paediatr. Dent..

[24] Nordeen K.A., Kharouf J.G., Mabry T.R., Dahlke W.O., Beiraghi S., Tasca A.W. (2022). Radiographic Evaluation of Permanent Second Molar Substitution After Extraction of Permanent First Molar: Identifying Predictors for Spontaneous Space Closure. Pediatr. Dent..

[25] Patel S., Ashley P., Noar J. (2017). Radiographic prognostic factors determining spontaneous space closure after loss of the permanent first molar. Am. J. Orthod. Dentofac. Orthop..

[26] Sabbagh H.J., Samara A.A., Agou S.H., Turkistani J., Al Malik M.I., Alotaibi H.A., Alsolami A.S.D., Bamashmous N.O. (2024). Spontaneous space closure after extraction of young first permanent molar. Retrospective cohort study. PeerJ.

[27] Serindere G., Bolgul B., Parlar T., Cosgun A. (2019). Effects of first permanent molar extracton on space changes observed in the dental arch using data mining method. Niger. J. Clin. Pract..

[28] Teo T.K., Ashley P.F., Derrick D. (2016). Lower first permanent molars: Developing better predictors of spontaneous space closure. Eur. J. Orthod..

[29] Elhussein M., Jamal H. (2020). Molar Incisor Hypomineralisation-To Extract or to Restore beyond the Optimal Age?. Children.

[30] Sabri R. (2021). Multidisciplinary management of permanent first molar extractions. Am. J. Orthod. Dentofac. Orthop..

[31] Saber A.M., Altoukhi D.H., Horaib M.F., El-Housseiny A.A., Alamoudi N.M., Sabbagh H.J. (2018). Consequences of early extraction of compromised first permanent molar: A systematic review. BMC Oral. Health.

[32] Hamza B., Papageorgiou S.N., Patcas R., Schatzle M. (2024). Spontaneous space closure after extraction of permanent first molars in children and adolescents: A systematic review and meta-analysis. Eur. J. Orthod..

[33] Eichenberger M., Erb J., Zwahlen M., Schatzle M. (2015). The timing of extraction of non-restorable first permanent molars: A systematic review. Eur. J. Paediatr. Dent..

[34] Alqanas S., Alsahiem J., Aljami A., Alsudairi N., Ahmad S., Sharma S., Rajinder S., Alamri A., Alhazmi H., Hegazi F. (2024). Factors related to spontaneous space closure following early first permanent molar extraction: A systematic review. Int. J. Paediatr. Dent..

[35] Wehrbein H. (1991). Problems in orthodontic space closure after loss of first permanent molars. Prakt. Kieferorthop..

[36] Jacobs C., Jacobs-Müller C., Luley C., Erbe C., Wehrbein H. (2011). Orthodontic space closure after first molar extraction without skeletal anchorage. J. Orofac. Orthop. Fortschritte Kieferorthopadie.

[37] Raveli T.B., Shintcovsk R.L., Knop L.A.H., Sampaio L.P., Raveli D.B. (2017). Orthodontic Replacement of Lost Permanent Molar with Neighbor Molar: A Six-Year Follow-Up. Case Rep. Dent..

[38] Cardoso P.C., Mecenas P., Normando D. (2022). The impact of the loss of first permanent molars on the duration of treatment in patients treated with orthodontic space closure and without skeletal anchorage. Prog. Orthod..

[39] Langer L.J., Pandis N., Mang de la Rosa M.R., Jost-Brinkmann P.G., Bartzela T.N. (2023). Eruption Pattern of Third Molars in Orthodontic Patients Treated with First Permanent Molar Extraction: A Longitudinal Retrospective Evaluation. J. Clin. Med..

[40] van der Bilt A., Engelen L., Pereira L.J., van der Glas H.W., Abbink J.H. (2006). Oral physiology and mastication. Physiol. Behav..

[41] Gaviao M.B., Raymundo V.G., Sobrinho L.C. (2001). Masticatory efficiency in children with primary dentition. Pediatr. Dent..

[42] Toro A., Buschang P.H., Throckmorton G., Roldan S. (2006). Masticatory performance in children and adolescents with Class I and II malocclusions. Eur. J. Orthod..

[43] McGrath C., Broder H., Wilson-Genderson M. (2004). Assessing the impact of oral health on the life quality of children: Implications for research and practice. Community Dent. Oral. Epidemiol..

[44] Omara M., Stamm T., Bekes K. (2021). Four-dimensional oral health-related quality of life impact in children: A systematic review. J. Oral. Rehabil..

[45] Culler C.S., Gunarajasingam D., Henshaw M.M. (2021). Preschool oral health-related quality of life: A practical guide to measurement tools. J. Public. Health Dent..

[46] Yang C., Crystal Y.O., Ruff R.R., Veitz-Keenan A., McGowan R.C., Niederman R. (2020). Quality Appraisal of Child Oral Health-Related Quality of Life Measures: A Scoping Review. JDR Clin. Trans. Res..

[47] Ahmed S., Berzon R.A., Revicki D.A., Lenderking W.R., Moinpour C.M., Basch E., Reeve B.B., Wu A.W., International Society for Quality of Life Research (2012). The use of patient-reported outcomes (PRO) within comparative effectiveness research: Implications for clinical practice and health care policy. Med. Care.

[48] Basch E. (2010). The missing voice of patients in drug-safety reporting. N. Engl. J. Med..

[49] Sperti E., Di Maio M. (2017). Outcomes research: Integrating PROs into the clinic—Overall survival benefit or not, it’s worth the trouble. Nat. Rev. Clin. Oncol..

